# Location matters: LAG3 levels are lower in renal cell carcinoma metastatic sites compared to primary tumors, and expression at metastatic sites only may have prognostic importance

**DOI:** 10.3389/fonc.2022.990367

**Published:** 2022-10-13

**Authors:** David A. Schoenfeld, Ross D. Merkin, Myrto Moutafi, Sandra Martinez, Adebowale Adeniran, Deepika Kumar, Lucia Jilaveanu, Michael Hurwitz, David L. Rimm, Harriet M. Kluger

**Affiliations:** ^1^ Section of Medical Oncology, Yale School of Medicine, New Haven, CT, United States; ^2^ Department of Pathology, Yale School of Medicine, New Haven, CT, United States

**Keywords:** LAG3, RCC, metastases, immunotherapy, biomarker

## Abstract

While great strides have been made in the treatment of advanced renal cell carcinoma (RCC) with the emergence of immune checkpoint inhibitors (ICIs) and VEGFR-targeting drugs, sizable proportions of patients still do not respond to upfront therapy and long-term responses only occur in a minority of patients. There is therefore a great need for the development of better predictors of response and an increased understanding of mechanisms of resistance to these therapies. Alternative immune checkpoints outside the PD-1/PD-L1 axis, such as LAG3, have been implicated as one mechanism of resistance to ICIs. These checkpoints thus represent attractive therapeutic targets, and indeed the LAG3 inhibitor relatlimab was recently approved for the treatment of metastatic melanoma in combination with anti-PD-1 therapy. LAG3 inhibitors are being evaluated for RCC as well. In this context, a better understanding of LAG3 expression patterns in RCC and how they relate to clinicopathologic features of disease and response to immunotherapy may give insight into mechanisms of resistance to PD-1 inhibitors and aid in the identification of subgroups of patients more likely to benefit from certain drug regimens. In this study, we assessed LAG3 protein levels in leukocytes in normal kidney adjacent to RCC, primary RCC tumors, and matched metastatic tumors, including large numbers of brain metastases. We found that LAG3 protein levels are on average lower at metastatic sites compared to matched primary tumors, and that the difference was more pronounced in patients with high-risk clinical characteristics, including those with larger primary tumor size, grade 4 tumors, IMDC poor-risk disease, and initial presentation with brain metastases. We further saw that the prognostic value of LAG3 levels varies depending on the tissue site queried (*i.e.*, primary tumor versus metastases), and that relatively higher LAG3 levels at metastatic sites may predict a better response to immunotherapy and longer overall survival after the development of metastatic disease. These findings may have important implications for the design of future studies involving LAG3 or other immunotherapies in RCC.

## Introduction

Over the past few years, the treatment paradigm for advanced renal cell carcinoma (aRCC) has shifted rapidly, with the emergence of immune checkpoint inhibitors (ICIs) that target CTLA-4 and PD-1/PD-L1, as well as newer-generation VEGFR-targeting drugs, such as cabozantinib and lenvatinib. Combination regimens consisting of dual ICIs targeting CTLA-4 and PD-1, or anti-PD-1/PD-L1 plus a newer-generation VEGFR inhibitor, have significantly extended survival compared to older therapies, leading to numerous new drug approvals (1–5). While the approval of these regimens represents a great advance in the treatment of aRCC, a sizable proportion of patients still do not respond to upfront therapy. In addition, long-term responses only occur in a minority of patients, and most patients will ultimately have disease progression (6).

Much prior work has explored mechanisms of primary and acquired resistance to ICIs, including in RCC (7–10). Loss of the MHC I complex and/or dysfunction in antigen processing and presentation, defects in T cell cytolytic signaling pathways, expansion of T regulatory cells and pro-tumor myeloid cell populations, and increased expression of immunosuppressive cytokines/chemokines are just some of the many ways that tumors overcome ICIs. Other immune checkpoints beyond PD-1 and CTLA-4, including lymphocyte activation gene-3 (LAG3), T cell immunoglobulin and mucin domain-containing protein 3 (TIM-3), and T cell immunoreceptor with Ig and ITIM domains (TIGIT), have also been implicated in ICI resistance (7, 11). Recent work using single-cell RNA sequencing (scRNA-seq) has further shown that more advanced stages of RCC are more highly enriched with exhausted CD8+ T cell populations, characterized by high expression of multiple immune co-inhibitory molecules, including PD-1, CTLA-4, LAG3, TIM-3, and TIGIT, as well as other markers of terminal T cell exhaustion, as part of a dysfunctional immune circuit that develops as RCC progresses to more advanced stages (12).

The alternative immune checkpoints LAG3, TIM-3, and TIGIT thus represent attractive therapeutic targets. Indeed, relatlimab, a first-in-class LAG3 inhibitor, was recently approved for the first-line treatment of metastatic melanoma in combination with the anti-PD-1 antibody nivolumab, improving progression-free survival and response rates compared to anti-PD-1 alone. Relatlimab has also demonstrated activity in patients with melanoma that relapsed or progressed on prior ICIs (13, 14). Extension of relatlimab and other LAG3 inhibitors in development to the treatment of ICI-resistant aRCC is a promising avenue of exploration (15). To that end, numerous clinical trials are currently underway testing LAG3 inhibitors in RCC, as summarized in **Table 1**.

**Table 1:** LAG3 clinical trials involving patients with RCC.

Study ID	Drug	Target	Sponsor	Phase	Diseases	Status	Details
NCT05148546	Relatlimab	LAG3	The Netherlands Cancer Institute	II	Primary, resectable, intermediate to high risk, clear-cell RCC	Enrolling	Three-arm phase II trial investigating different neoadjuvant immunotherapy regimens, including nivolumab alone, ipilumamab + nivolumab, or ipilumamab + nivolumab
NCT03538028	INCAGN02385	LAG3	Incyte Biosciences International Sàrl	I	RCC plus multiple others	Completed	Phase I study to determine the safety, tolerability, and preliminary efficacy of INCAGNO2385
NCT03849469	XmAb22841	CTLA-4 and LAG3 bispecific	Xencor,Inc.	I	RCC plus multiple others	Active but not enrolling	Phase 1, multiple dose, ascending-dose escalation study and expansion study of XmAb22841 monotherapy and in combination with pembrolizumab (DUET-4)
NCT03005782	REGN3767	LAG3	Regeneron Pharmaceuticals,Inc.	I	Advanced malignancies	Enrolling	Study to evaluate safety and pharmacokinetics of REGN3767 as monotherapy and in combination with cemiplimab (anti- PD-1) in patients with advanced malignancies, including lymphoma.
NCT04626479	Favezelimab	LAG3	Merck Sharp & Dohme LLC	I/II	First-line RCC	Enrolling	Substudy 03A of umbrella study U03 to test experimental combinations of investigational agents in participants with first line RCC; one arm studies favezelimab + pembrolizumab + lenvatinib
NCT04626518	Favezelimab	LAG3	Merck Sharp & Dohme LLC	I/II	Second-line plus RCC	Enrolling	Substudy 03B of umbrella study U03 to test experimental combinations of investigational agents in participants with advanced second line plus RCC; one arm studies favezelimab + pembrilizumab
NCT05347212	Relatlimab	LAG3	MD Anderson Cancer Center	II	Advanced renaI medullary carcinoma	Approved	Study to test efficacy and safety of relatlimab +nivolumab in advanced renal medullary carcinoma
NCT03335640	Relatlimab	LAG3	Bristol-Myers Squibb	I	Advanced solid tumors treated with prior therapy	Active but not enrolling	Study to evaluate the treatment of solid tumors with various immunotherapy combinations based upon a broad biomarker- assessment.

Much effort has also been devoted to the development of robust predictive and prognostic biomarkers for ICIs. Currently, PD-L1 levels and tumor mutational burden are the most established, albeit imperfect, of these across various tumor types (16, 17). In RCC, neither has much predictive value and they are not routinely used in clinical practice to guide treatment choice (18, 19). Additionally, unlike for other tumor types, in RCC higher CD8+ T cell infiltration is associated with a worse overall prognosis, although T cell infiltration patterns don’t appear predictive of ICI-responsiveness (20, 21). However, high baseline myeloid inflammation has been associated with worse outcomes to anti-PD-1 therapy (22). In contrast, the presence of *PBRM1* mutations has been proposed as a positive predictive factor for response to ICIs in RCC (23, 24). Additionally, a recent single-cell transcriptomic analysis of RCC patients pre- and post-ICI treatment found that ICI-responders displayed more intra-tumoral T cell differentiation towards terminally differentiated states, potentially derived from low-abundance progenitor-exhausted T cells (25). Immune checkpoint expression was upregulated in these T cells, and the authors also found immunosuppressive transcriptional programs in tumor-associated macrophages and cancer cells in ICI-responders, all potential mechanisms of eventual resistance. Additional efforts using an integrated multi-omics approach identified RCC molecular subsets with differing sensitivities to anti-angiogenic therapy or ICIs (26).

Predictive and prognostic biomarkers will be similarly needed for the newer ICIs in development targeting LAG3, TIM-3, and TIGIT. Ideally, these biomarkers will aid in generating optimal combination regimens and guide the sequencing of therapies. In anticipation of the emergence of these newer drugs, a recent study by Takamatsu et al. looked at the expression of LAG3, TIM-3, and TIGIT in primary RCC and in metastases to various anatomic locations, including four brain metastases (27). LAG3, TIM-3, and TIGIT expression seemed to be mutually exclusive within the tumor microenvironment and could be used to be define distinct tumor subtypes. In primary tumors, the LAG3 subtype was associated with a worse prognosis and a more immunosuppressive microenvironment, as defined by higher infiltration with exhausted T cells and tumor associated macrophages. The LAG3 subtype also had higher exhausted T cell levels in metastases compared to the TIM-3 and TIGIT subtypes.

By profiling samples from distinct anatomic locations, Takamatsu et al. tried to account for the high degree of inter-lesional heterogeneity that defines RCC (28, 29). RCC is also characterized by high intra-lesional heterogeneity. Taken together, these factors make studies reliant on single-site biopsies from patients potentially subject to sampling bias and not fully representative of disease states. For example, a prior study from our group assessed PD-L1 levels using quantitative immunofluorescence in matched pairs of nephrectomy and metastatic sites on tissue microarrays (TMAs), with four biopsy cores used for each tumor specimen, and found a weak correlation in expression between primary and metastatic sites, as well as intra-tumor heterogeneity at all sites (30). Similar findings were observed with tumor-based drug targets (31–33).

In this study, we assessed LAG3 expression in tumor infiltrating leukocytes on matched normal kidney and primary RCC cases, and matched primary and metastatic RCC cases, including brain metastases. LAG3 expression was higher in primary than matched metastatic sites, and higher levels in metastatic sites were associated with improved response to immunotherapy.

## Materials and methods

### Tissue microarrays

We performed these studies using three different RCC TMAs. Two have been previously described (31, 34): Yale TMA-84 (YTMA-84), composed of matched adjacent normal kidney and primary tumor pairs from renal tumors resected between 1987 and 1999; and Yale TMA-166 (YTMA-166), composed of matched primary tumor and metastasis pairs (from separate RCC patients than YTMA-84) with metachronous or synchronous metastatic disease treated between 1978 and 2011, with four 0.6 mm cores, or “replicates,” from different areas of each tumor specimen within the same formalin-fixed, paraffin-embedded (FFPE) block, placed in two TMA blocks. During the creation of these TMAs, two independent pathologists reviewed and selected areas of tumor and adjacent normal kidney, when appropriate. Due to usage of these TMAs since their construction, there was some depletion of tissue cores – only intact tissue cores were subject to LAG3 expression assessment.

We also constructed a new RCC TMA from archived, FFPE tumor specimens exclusively from patients who developed brain metastases (Yale TMA-528; YTMA-528). These patients were identified using clinical databases, and available tumor specimens, from all tumor sites including primary tumors and brain and non-brain metastases, were collected. Resections or biopsies were performed between 2002 and 2021. Hematoxylin and eosin–stained slides were reviewed by two independent pathologists (AA and DK) and representative tumor areas were selected. Two 0.6 mm cores were extracted per tumor block, as allowed by the amount of tumor material, and used to construct two master TMA blocks. For each block, serial 5 *μ*m sections were cut and placed on slides. An extensive clinical database was constructed based on the specimens included in the TMA, and censoring of clinical data occurred on 5/1/2022. Survival and response to immunotherapy analysis were based on the patients included in the TMA and this database, which included clinical follow-up data. Pathologic grades of specimens were based on the Fuhrman grading system as scored by an accredited pathologist at the time of biopsy, with a transition to the WHO/ISUP system in 2021. Representative tumor spots of grades 1-4 are shown in **Supplementary Figure S1**. Specimens and clinical information were collected with the approval of a Yale University Institutional Review Board.

### Digital spatial profiling

To determine LAG3 expression in tumor infiltrating leukocytes, we performed digital spatial profiling (DSP) on a GeoMx DSP instrument (NanoString Technologies) according to the manufacturer’s instructions and based on previously described methods (35). Briefly, TMA slides were deparaffinized and subjected to antigen retrieval. They were then stained with three fluorescently-labeled antibodies to define the cellular compartments: macrophages by CD68; leukocytes by CD45; and tumor cells by pan-cytokeratin. After this, they were incubated with a panel of photocleavable oligonucleotide-conjugated antibodies validated by NanoString and directed towards immuno-oncology markers, including LAG3, as well as three housekeeping proteins (GAPDH, histone H3, ribosomal protein S6) and three negative controls (mouse IgG1, mouse IgG2a and rabbit IgG). The slides were subsequently loaded onto the GeoMx DSP instrument and digitally scanned to produce fluorescent images of the cellular compartments. Individual tissue cores or “spots” were visually inspected and regions of interest covering the entire intact core, with a maximum diameter of 660 μm, were specified. For YTMA-84, only tumor spots with an intact matched adjacent normal kidney spot were analyzed, and each tissue specimen had *n=*1. For YTMA-166, all intact tumor cores with at least one matched sample (primary tumor to metastases) were analyzed, including all replicates. For YTMA-528, all intact cores were analyzed. Each region of interest was further divided into cellular-molecular compartments based on fluorescence patterns, to be collected based on the following hierarchy: macrophage compartment (CD68+); leukocyte compartment (CD68-CD45+); and tumor compartment (pan-cytokeratin+). Images of two representative spots, including a hematoxylin and eosin stain, the fluorescence patterns of the cellular-molecular compartment markers, and the compartment masks created by the GeoMx instrument, are shown in **Supplementary Figure S2**.

The cellular-molecular compartments for each region of interest were sequentially illuminated with ultraviolet light to cleave the oligonucleotide from the anti-LAG3 antibody. The oligonucleotides were collected by microcapillary aspiration and deposited into wells of a 96-well plate. They were then hybridized to four-color, six-spot optical barcodes and quantitated using the nCounter platform (NanoString Technologies). Quality control checks as specified by the manufacturer were performed and digital counts were first normalized to internal spike-in controls (External RNA Control Consortium). They were then further normalized to the geometric mean of the levels of two housekeeping genes (histone H3 and ribosomal protein S6) within each compartment for a given region of interest.

### Statistical analysis

The quantified LAG3 protein counts within the leukocyte compartment generated by the nCounter platform were used for all downstream analysis. For YTMA-84 and YTMA-166, only patients with intact matched-pair samples were included in the analysis. For YTMA-528, only matched pairs of primary and metastatic tumors were included for some analysis, although all samples were included at times where specified. For YTMA-166 and -528, when there were multiple replicates for a given tumor specimen, the average normalized protein count was used. Matched-pair analysis was performed using the non-parametric Wilcoxon test. The non-parametric Kruskal-Wallis test was used to compared the means of three or more groups. For survival analysis, the median levels of LAG3 within a tumor type were used as the cut-point. Kaplan-Meier plots were generated, and log-rank tests were performed. Response to immunotherapy was determined retrospectively using Response Evaluation Criteria in Solid Tumors (RECIST) version 1.1, for patients whose tumors were included in YTMA-528. Overall response rate (ORR) was defined as the proportion of patients with a best overall response of complete or partial response. Disease control rate (DCR) was defined as the proportion of patients with a best response of complete response, partial response, or stable disease lasting at least six months. All statistical testing was performed with a two-sided *p* < 0.05 considered significant. GraphPad Prism for Windows software version 8.0 (GraphPad Software, Inc., La Jolla, CA) was used for data visualization and statistical testing.

## Results

Descriptive statistics for the three RCC TMAs analyzed in this study are shown in **Table 2**. For YTMA-84, 25 pairs of matched adjacent normal kidney: primary tumor, each tissue specimen from a single core (*n=*1), were analyzed. For YTMA-166, there were 14 matched primary tumor and metastases pairs. Nearly 80% of these tumor specimens had more than one core or “replicate”, with 64% having 3 or 4. Of note, ~40% of the metastases were from the lung or bone. For YTMA-528, 95 tumor specimens from 59 unique patients were analyzed, with 24 matched primary tumor and metastases pairs. 72% of the tumor specimens had two replicates. Brain metastases represented 25% of the total TMA; lung and bone metastases comprised approximately another one-third. Additionally, 69% of the patients included in this TMA had received an immunotherapy drug at some point in their treatment course, as summarized in **Table 3**. Of note, 95% of these regimens contained an anti-PD-1 agent, with 38% consisting of single-agent anti-PD-1 and another 31% comprising anti-PD-1 plus anti-CTLA-4.

**Table 2:** TMA descriptive statistics.

	n (%) or median (95% confidence interval)		
	YTMA-84	YTMA-166	YTMA-528
**Unique Patients**	25	14	59
**Unique Samples**	50	28	95
**Samples with replicate #:**			
* 1*	50 (100)	6 (21)	27 (28)
* 2*	0 (0)	4 (14)	68 (72)
* 3*	0 (0)	9 (32)	0 (0)
* 4*	0 (0)	9 (32)	0 (0)
**Matched pairs**	25	14	24
**Age, years (at sample date)**	71.0 (62.0,76.0)	56.0 (50.0,69.0)	60.6 (57.9,62.8)
**Male**	15 (60)	9 (64)	46 (78)
**Histology:**			
* ccRCC*	22 (88)	–	87 (92)
* nccRCC*	3 (12)	–	8 (8)
**IMDC score:**			
* Good*	–	–	6 (13)
* Intermediate*	–	–	29 (64)
* Poor*	–	–	10 (22)
**Nephrectomy**	–	–	50 (85)
**Primary tumor size (cm)**	5.0 (3.5, 7.0)	6.5 (3.5, 10.7)	9.8 (8.0, 11.0)
**Sample Location:**			
* Adjacent normal kidney*	25 (50)	0 (0)	0 (0)
* Primary*	25 (50)	14 (50)	28 (29)
* Metastatic - brain*	0 (0)	0 (0)	24 (25)
* Metastatic - lung*	0 (0)	4 (14)	19 (20)
* Metastatic - bone*	0 (0)	2 (7)	15 (16)
* Metastatic - other*	0 (0)	8 (29)	9 (9)
**Presence of *de novo* brain mets**	–	–	12 (20)
**Received IO therapy at some point**	–	–	41 (69)

**Table 3:** Immunotherapy regimens.

Immunotherapy agent	n (%)
any anti-PD-1	62 (95)
anti-PD-1 + anti-CTLA-4	20 (31)
anti-PD-1 + TKI	4 (6)
anti-PD-1 + other*	13 (20)
single-agent anti-PD-1	25 (38)
other immunotherapy**	3 (5)
Total***	65 (100)

*Other agents with anti-PD-1: bevacizumab; NKTR-214; IFN; IL-21; **Other immunotherapies: OX-40 agonist (2); single-agent ipilimumab (1); ***The total number of immunotherapy agents exceeds the total patient number (41) because patients often received more than one regimen. TKI, Tyrosine kinase inhibitor.

**Figure 1:**
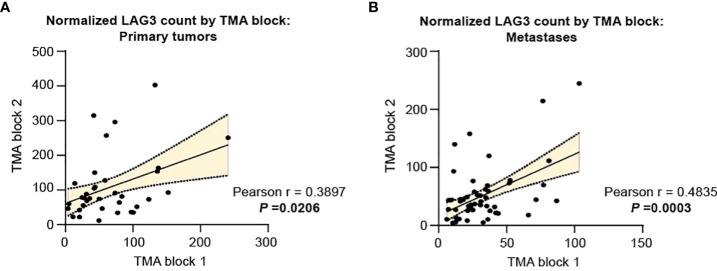
Intratumor heterogeneity in LAG3 expression is more pronounced in primary specimens compared to metastases. LAG3 expression in replicate cores from the same tumor specimen for **(A)** primary and **(B)** metastatic tumors. The linear best-fit line is shown with the 95% confidence band. The Pearson correlation coefficient (r) and the associated p-value were calculated for each plot.

Using these TMAs, we assessed LAG3 protein levels in the CD68-CD45+ leukocyte compartment. Considering the high degree of intra-tumoral heterogeneity present in RCC, we took advantage of the multiple replicates analyzed in TMAs-166 and -528 to first assess the correlation in LAG3 protein counts across replicate cores for the same tumor specimen. As shown in **Figure 1A**, within primary tumors there was a modest but statistically significant correlation in LAG3 protein expression between replicate cores (r=0.3897, *p*=0.0206), indicating that some degree of intra-tumoral heterogeneity in LAG3 expression was present. There was a higher degree of intra-tumor correlation across TMA blocks in the metastases (**Figure 1B**; r=0.4835, *p*=0.0003).

**Figure 2:**
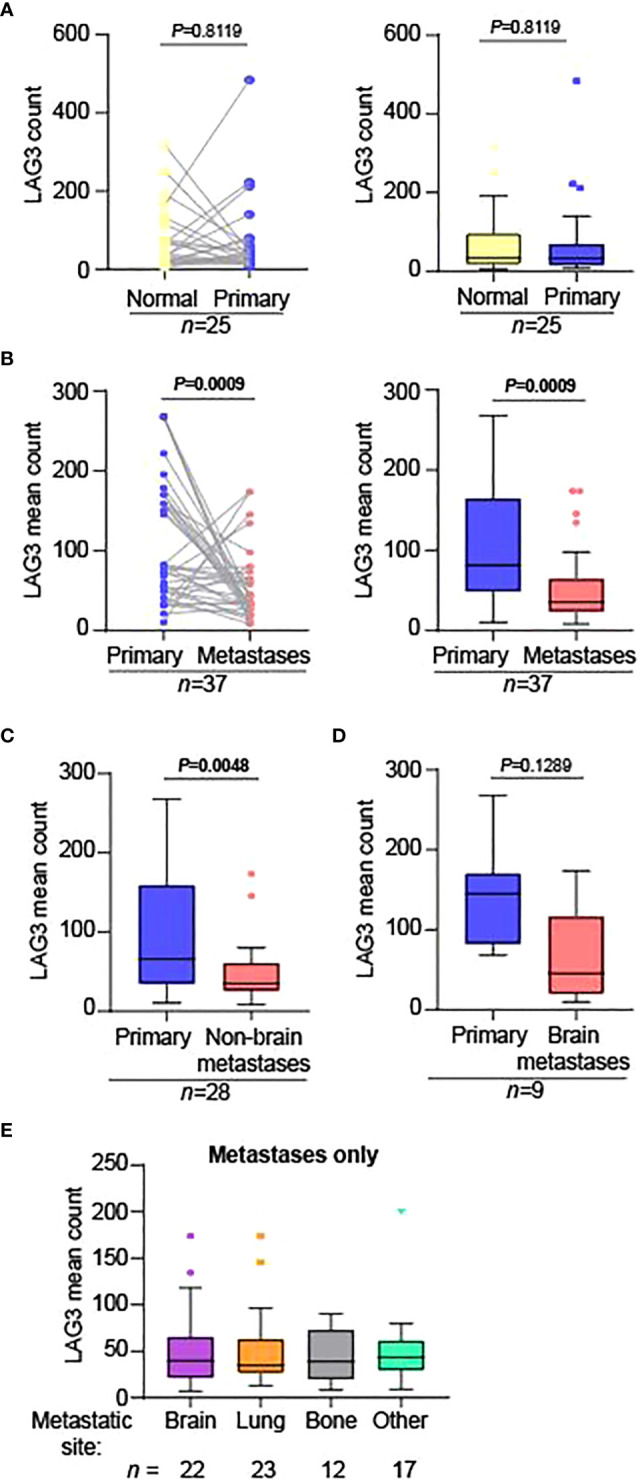
LAG3 levels are significantly lower in metastases compared to primary tumors. Matched-pair analysis comparing the normalized LAG3 counts in **(A)** normal adjacent kidney (“Normal”) to primary tumors (“Primary”) and **(B)** primary tumors to metastases. The graphs in the left and right panels represent the same data displayed differently; the graphs in the right panels are Tukey plots. Matched-pair analysis comparing the normalized mean LAG3 counts in primary tumors to **(C)** non-brain and **(D)** brain metastases separately. For parts **(B-D)**, when there were multiple metastatic specimens from the same patient on the TMA, they were each compared to the corresponding primary tumor separately. The n-sizes displayed represent the number of primary tumor to metastasis pairings, from **(B)** 28, **(C)** 22, and **(D)** 6 unique patients. **(E)** Tukey plot showing the normalized mean LAG3 counts across different anatomic sites of metastases only. There were no significant differences between the groups. For parts **(A-D)**, matched-pair analysis was performed using the non-parametric Wilcoxon test. For part **(E)**, the non-parametric Kruskal-Wallis test was performed comparing the means of all groups.

We next compared normalized LAG3 counts across matched-pair samples as tumor stage became progressively more advanced (normal kidney ➔ primary tumor; primary tumor ➔ metastases). LAG3 levels were not significantly different when comparing matched adjacent normal kidney to primary tumors (**Figure 2A**) but were significantly lower in the metastases compared to primary lesions (**Figure 2B**). This difference was maintained when comparing primary tumors to non-brain metastases only (**Figure 2C**), and there was a trend towards lower LAG3 levels in brain metastases, albeit non-significant (**Figure 2D**), potentially reflecting the lower n-size in this group (n=9). Supporting this, when LAG3 levels were compared across different anatomic sites of metastases only (brain, lung, bone, and other), there were no significant differences (**Figure 2E**).

**Figure 3:**
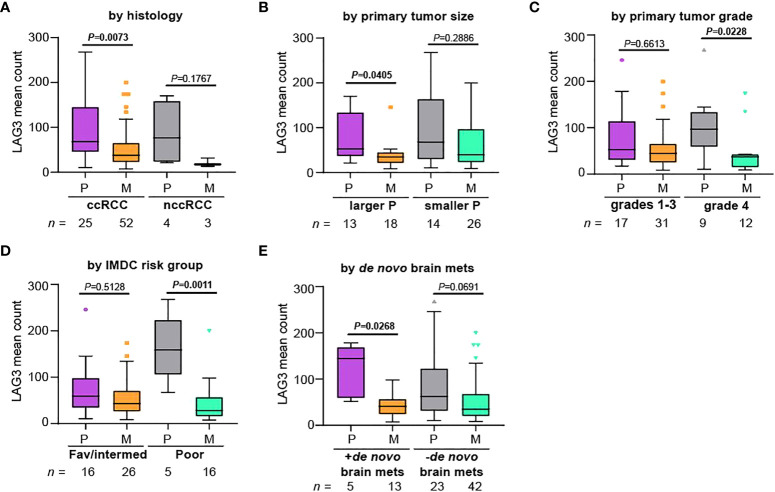
Differences in LAG3 expression between primary and metastatic tumors are more pronounced in patients with certain high-risk features. Patients were divided based on **(A)** histology, **(B)** primary tumor size, **(C)** primary tumor grade, **(D)** IMDC risk group, and **(E)** whether the patient had *de novo* brain metastases, and normalized mean LAG3 counts were compared between primary tumors (“P”) and metastases (“M”) within each group. We note that this cohort was enriched for patients with brain metastases. For **(B)**, patients were segmented based on the median primary tumor size. The Kruskal-Wallis test was performed comparing primary tumors to metastases. All graphs represent Tukey plots. ccRCC, clear cell RCC; nccRCC, non-clear cell RCC; IMDC, International Metastatic RCC Database Consortium; Fav/intermed, favorable/intermediate; brain mets, brain metastases.

We next asked if LAG3 expression differences between primary tumors and metastases were maintained across various pathologic and clinical variables. For this analysis, we only used YTMA-528, for which we had extensive pathologic and clinical data for each patient. Nearly all available tumor samples were of clear cell histology (**Table 2** and **Figure 3A**), and among clear cell RCC cases, LAG3 levels were significantly lower in metastases. Samples sizes were very small for specimens of non-clear cell histology, but LAG3 levels trended lower in metastases as well. When dividing the cases by the size of the primary tumors, only those with larger primary tumors had significantly lower LAG3 levels in metastases (**Figure 3B**). Likewise, compared to lower grade primary tumors (grades 1-3), only grade 4 tumors were associated with significantly lower LAG3 in metastases (**Figure 3C**). We also divided patients by their International Metastatic RCC Database Consortium (IMDC) risk score and found reduced LAG3 expression in metastases from “Poor” risk group patients, but not patients in the “Favorable” or “Intermediate” risk groups (**Figure 3D**). When segmenting patients by the presence or absence of brain metastases at the time of initial diagnosis of metastatic RCC, *i.e.*, *de novo* brain metastasis, the differential expression of LAG3 between primary tumors and metastases was only preserved in those with *de novo* brain metastases and not in patients who developed brain metastases later in the course of their illness (**Figure 3E**).

**Figure 4:**
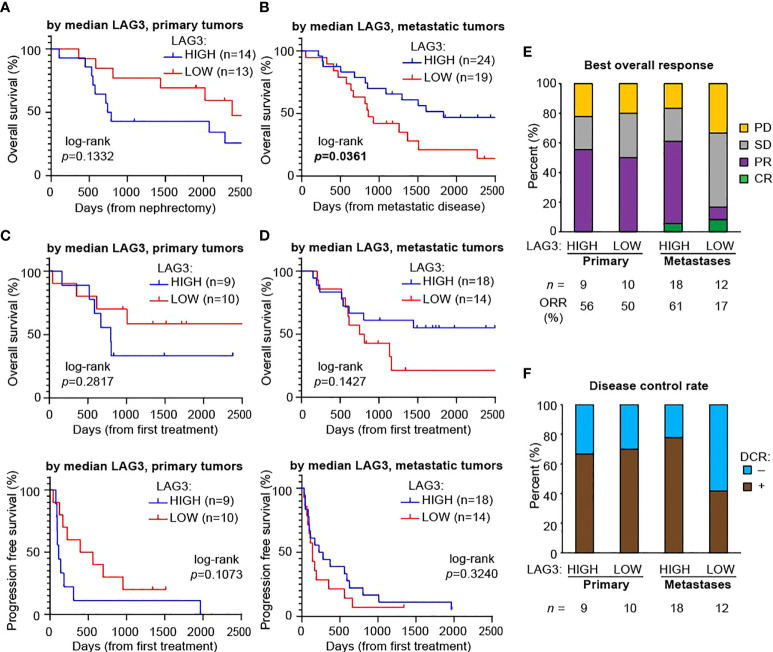
Higher LAG3 in metastases is associated with longer overall survival after developing metastatic disease and a higher response rate to immunotherapy. Kaplan-Meier plots of overall survival based on median LAG3 levels in **(A)** primary tumors after nephrectomy and **(B)** metastases after developing metastatic disease. Kaplan-Meier plots of overall survival (top panels) and progression-free survival (bottom panels) after the first immunotherapy drug administered based on median LAG3 levels in **(C)** primary tumors and **(D)** metastases. All graphs were cut-off at day 2,500 for visualization purposes, with no meaningful changes to the data after this point. Statistical testing was performed with the log-rank test. **(E)** Best overall responses to immunotherapy. Cohort sizes and the overall response rate (ORR), defined as the proportion of patients with a best overall response of complete or partial response, are displayed beneath the graph. **(F)** Disease control rates (DCR), defined as the proportion of patients with a best response of complete response, partial response, or stable disease lasting at least six months. Cohort sizes are displayed beneath the graph. CR, complete response; PR, partial response; SD, stable disease; PD, progressive disease.

We next explored whether LAG3 levels had prognostic value and whether this depended on the tissue site queried (*i.e.*, primary tumor versus metastases). We divided patients included on YTMA-528 into “HIGH” or “LOW” LAG3 expressors based on the overall median expression, looking separately in primary tumors (median LAG3 count=67.5) and then metastases (median LAG3 count=35.6). We then analyzed overall survival (OS) from the time of nephrectomy for the primary samples and from the time of development of metastatic disease for the metastatic samples. LAG3 expression in primary tumors was not associated with OS (log-rank *p =* 0.1332; **Figure 4A**). Conversely, in metastases, higher LAG3 expression was associated with improved OS (log-rank *p =* 0.0361; **Figure 4B**).

Since over two-thirds of the patients included on YTMA-528 had also received at least one immunotherapy drug at some point, we also looked for an association between LAG3 expression and both OS and progression free survival (PFS) with the first immunotherapy drug. For lower LAG3 expression (dichotomized by median expression) in primary tumors there was a trend towards better OS and PFS using the first immunotherapy treatment as the starting timepoint, although this did not reach statistical significance (**Figure 4C**). LAG3 expression in metastases was similarly not significantly associated with OS and PFS, although there was a weak trend towards better OS in patients with higher LAG3 (**Figure 4D**). We next determined the best overall response to immunotherapy treatment among these patients (**Figure 4E**). Based on expression in primary tumors, the ORR (complete response + partial response) was similar in high versus low LAG3 expressors (56% versus 50%). However, in metastases, patients whose tumors had high LAG3 expression had nearly a four-fold increase in ORR (61% versus 17%). Similar trends were evident when looking at DCR (complete response + partial response + stable disease for > 6 months): in primary tumors, rates were comparable regardless of LAG3 expression, while in metastases, the disease control rate was nearly twice as high in patients whose metastases had high LAG3 expression (**Figure 4F**).

## Discussion

In this study, we assessed LAG3 expression in large numbers of RCC samples. We did not see major differences in LAG3 expression in primary RCC tumors compared to their adjacent normal kidney. However, when comparing matched primary tumors and metastases, LAG3 levels were significantly lower in the metastases. Differences between primary and metastatic sites were more pronounced in patients with higher-risk characteristics, including larger primary tumor size, higher grade, IMDC poor-risk disease, and the presence of brain metastasis at initial diagnosis.

The lower average LAG3 protein levels in metastatic sites compared to primary tumors need to be cautiously interpreted in the context of findings from Braun *et al*, which showed that more advanced stages of RCC are more highly enriched with exhausted CD8+ T cell populations, characterized by high expression of multiple immune checkpoints, including LAG3 (12). Pseudo-time analysis from this study indeed found that LAG3 transcript levels increased as RCC became progressively more advanced. Some potential explanations for the differing results include: our quantification of protein versus RNA when using scRNA-seq; our use of a technology that groups cells of a specific type compared to scRNA-seq which has single cell resolution; and our looking in a mixed non-macrophage leukocyte population (CD68-CD45+) versus a pure T cell population in the Braun et al. study. Differences in cohort sizes and the use of matched patient pairs could also account for some of the differences: the Braun et al. study encompassed 13 total unmatched tumor specimens, along with adjacent normal tissue, whereas our study consisted of 25 matched primary tumors and adjacent normal kidney specimens, and 37 matched primary tumor and metastases pairs, most with multiple replicates. Of note, as we cannot assess protein levels on a single-cell basis in our study, it is also possible that the LAG3 intensity per individual cell might be higher among certain cellular subsets in metastases compared to primary sites but lower on aggregate. Our findings could also be consistent with an increase in progenitor exhausted T cells, which have lower expression of immune checkpoints compared to terminally exhausted T cells (36–38). Future studies using DSP to assess mRNA levels might enable us to understand the differences seen. Our inclusion of YTMA-528 and samples exclusively from patients who developed brain metastases may have also led to different results. For example, in previous studies of melanoma brain metastases compared to matched metastatic samples, we found that PD-L1 expression and T cell content was globally lower in the brain, indicating that the tumor microenvironment in the brain might differ from other anatomic sites (39, 40).

As noted previously, RCC is characterized by a high degree of intra- and inter-tumoral heterogeneity, and studies that rely on single-site, single-tumor biopsies may have inherent biases or may not fully reflect disease phenotypes. In the TMAs containing matched tumor pairs, most of our tumor specimens had at least two replicate samples from nearby tumor areas. While still statistically significant, there was only a modest correlation in LAG3 protein counts across replicate cores for the primary tumors, seeming to validate our strategy. This finding is similar to that for PD-L1 levels as assessed by quantitative immunofluorescence in prior work using one of the TMAs analyzed in this study (YTMA-166) (30). There was a slightly stronger correlation among replicate cores in the metastases. These findings have important clinical implications and suggest that if LAG3 expression is used for future predictive purposes for patient selection, more than one biopsy might be indicated, particularly for primary tumors, as a negative result might unnecessarily exclude patients from LAG3 targeting therapy. However, we did not see significant differences in LAG3 expression across different anatomic sites of metastases, including in the brain, indicating that this factor may not be as relevant when selecting a site to biopsy.

Having seen that differences in LAG3 levels between primary tumors and metastases were more pronounced in patients with higher risk features, we asked whether LAG3 levels had prognostic value in our datasets. At primary tumor locations, there was no clear association between LAG3 in the primary tumor and OS after nephrectomy. At metastatic sites, however, higher LAG3 levels were associated with improved OS after developing metastases, although there was no clear association with survival after receiving immunotherapy. However, when we dichotomized samples by the median LAG3 expression within each anatomic location, we saw that high LAG3 expression in the metastatic samples was associated with nearly four-times higher ORR and nearly twice the DCR from immunotherapy compared to low LAG3 expression. Among primary samples, there was no such association, and as LAG3 is developed as a predictive biomarker, levels should be interpreted in the context of the disease being treated; patients treated after nephrectomy might be better assessed by LAG3 levels in metastatic sites rather than archival nephrectomy tissue.

Overall, these data suggest that the predictive and prognostic significance of LAG3 levels may be dependent on biopsy location, and that higher LAG3 levels at metastatic sites only may be associated with longer overall survival and better response to immunotherapy. They also indicate that LAG3 levels at metastatic sites may be a biomarker for response to immunotherapies as a class in general, as our patient population had received a mix of immunotherapy regimens, although nearly all contained an anti-PD-1 agent, either alone or in combination. Although it would stand to reason that LAG3 levels would at least be an equal biomarker for response to anti-LAG3 therapies, we cannot draw conclusions in this regard from our data, as no patients in our cohort were treated with anti-LAG3 antibody therapies. Our results are also based on a quantitative assessment of protein levels within a particular cellular compartment, whereas the biomarkers widely used today that assess protein levels generally rely on cell positivity measures (41). Still, our findings demonstrate that the type of tumor tissue being evaluated may have large effects on the prognostic and predictive power of certain biomarkers, including LAG3.

This study has some notable weaknesses. The technology used does not have single cell resolution, and so it is unclear if certain subsets of cells within the CD68-CD45+ compartment are driving the changes in LAG3 expression. Our survival analysis was based on subgroups with small cohort sizes and thus had limited power. We also based our response assessment for immunotherapy-treated patients on a heterogenous group of patients that had received various immunotherapy regimens. While LAG3 levels still correlated with differences in response rates, it is unclear how this would apply to distinct treatment regimens.

In summary, we assessed LAG3 protein levels in the non-macrophage immune compartment (CD68-CD45+) in a large cohort of matched-pair RCC tissue specimens, often containing multiple replicates per specimen. We found that LAG3 levels were lower in metastases compared to primary tumors, and that patients with certain high-risk features had more significant differences in LAG3 expression between primary and metastatic sites. Intra-tumor heterogeneity was seen in both primary and metastatic sites, indicating that if LAG3 expression is used for predictive purposes, more than one biopsy may be indicated. Moreover, within-patient differences between primary and metastatic sites suggest that for the treatment of metastatic disease, primary tumor tissue might not be sufficiently informative. Higher expression of LAG3 in metastases was associated with longer overall survival and predicted a better response to immunotherapy treatments. These results may have important implications for the design of future studies involving LAG3 inhibitors or other immunotherapies in RCC.

## Data availability statement

The raw data supporting the conclusions of this article will be made available by the authors, without undue reservation.

## Ethics statement

This study was reviewed and approved by Yale University Institutional Review Board. The patients/participants provided their written informed consent to participate in this study.

## Author contributions

Conceptualization: DS, HK. Methodology and Investigation: DS, RM, MM, SM, AA, DK, LJ, MH. Funding acquisition: HK. Supervision: DR, HK. Writing: DS, RM, HK All authors contributed to the article and approved the submitted version.

## Funding

The work was funded in part by NIH grants P50 CA121974 (M. Bosenberg and HK), R01 CA227472 (HK and K. Herold), R01 CA216846 (HK and G. Desir), and T32-CA233414-02 (DS).

## Acknowledgments

The authors would like to acknowledge Lori Charette and Yale Pathology Tissue Services for help in constructing YTMA-528.

## Conflict of interest

HK has received consulting fees from Iovance, Immunocore, Celldex, Merck, Elevate Bio, Instil Bio, Bristol-Myers Squibb, Clinigen, Shionogi, Chemocentryx, Calithera, Signatero, Gigagen, GI Reviewers, all outside of the submitted work. HK has also received research grant funding (to Yale University) from Merck, Bristol-Myers Squibb and Apexigen. MH reports - Advisory Boards: Bristol Myer Squibb, CRISPR Therapeutics, Exelixis, Nektar Therapeutics, Janssen. Research: Alpine, Achilles Therapeutics, Apexigen, Arrowhead, Astellas, AstraZeneca, Bayer, Bristol Myer Squibb, CRISPR Therapeutics, Corvus, Eli Lilly, Endocyte, Fate Therapeutics, Genentech, Genmab, GSK, Innocrin, Iovance, KSQ, Merck, Nektar Therapeutics, Novartis, Pfizer, Progenics, Sanofi Aventis, Seattle Genetics, Tmunity, Torque, Unum. Other: Gamida Cell, Arvinas. DR reports - Advisory boards: Amgen, Astra Zeneca, Cell Signaling Technology, Cepheid, Danaher, GSK, Konica/Minolta, Lilly, Merck, Monopteros, Nanostring, PAIGE.AI, Regeneron, Roche, Ventana. Consultant: Fluidigm, Immunogen, NextCure, Odonate, Sanofi, Verily. Research support: Amgen, Cepheid, Navigate BioPharma, NextCure, Konica/Minolta. Instrument support: Akoya. Royalty: Rarecyte. SM is currently an employee at Boehringer Ingelheim, Inc.

The remaining authors declare that the research was conducted in the absence of any commercial or financial relationships that could be construed as a potential conflict of interest.

## Publisher’s note

All claims expressed in this article are solely those of the authors and do not necessarily represent those of their affiliated organizations, or those of the publisher, the editors and the reviewers. Any product that may be evaluated in this article, or claim that may be made by its manufacturer, is not guaranteed or endorsed by the publisher.
