# Supplementary figures and images for: Location matters: LAG3 levels are lower in renal cell carcinoma metastatic sites compared to primary tumors, and expression at metastatic sites only may have prognostic importance
Source: Front Oncol. 2022 Oct 13;12:990367. doi: 10.3389/fonc.2022.990367 (PMC9608089; doi:10.3389/fonc.2022.990367)

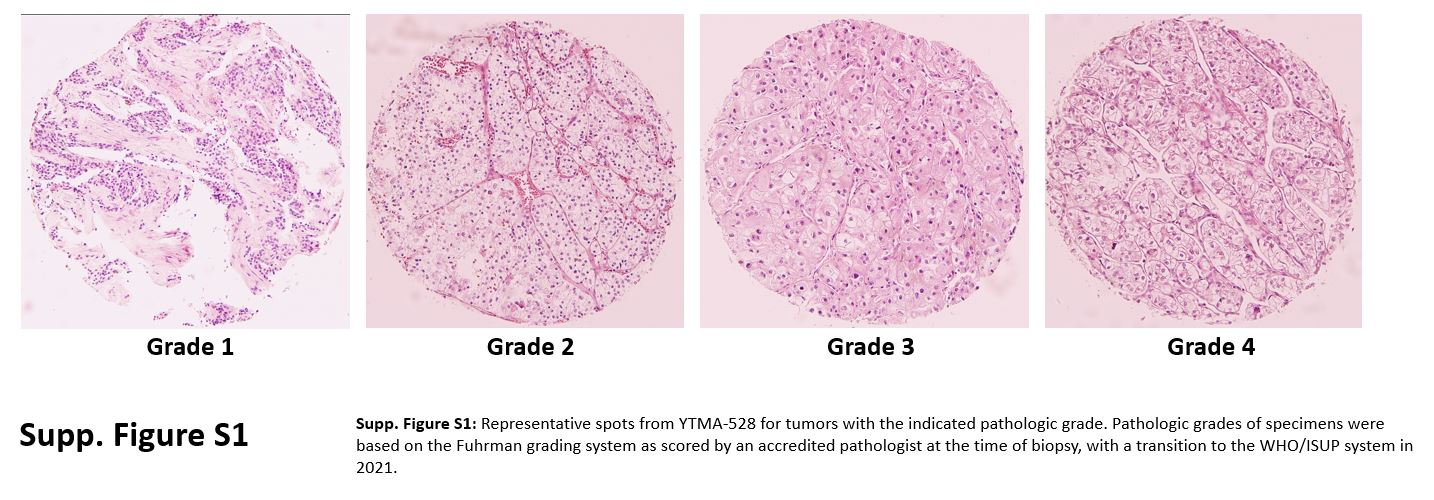

Supplement: Supplementary file 1 [file Image_1.jpeg]

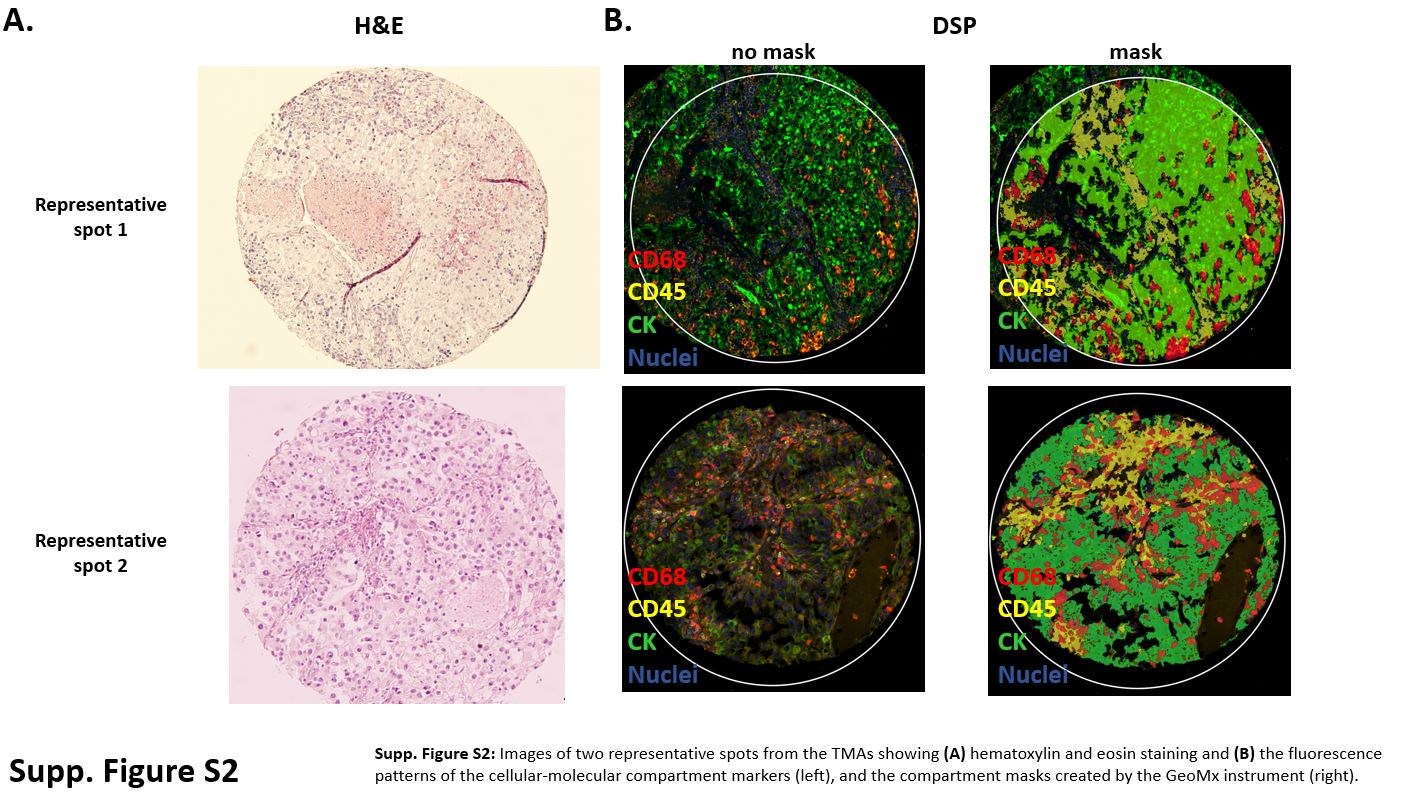

Supplement: Supplementary file 2 [file Image_2.jpeg]
